# Resistive switching studies in VO_2_ thin films

**DOI:** 10.1038/s41598-020-60373-z

**Published:** 2020-02-24

**Authors:** Abhimanyu Rana, Chuan Li, Gertjan Koster, Hans Hilgenkamp

**Affiliations:** 1 Faculty of Science and Technology, and MESA+ Institute of Nanotechnology, University of Twente, Enschede, The Netherlands; 2 School of Engineering and Technology, BML Munjal University, Gurgaon, India

**Keywords:** Electronic devices, Electronic properties and materials

## Abstract

The hysteretic insulator-to-metal transition of VO_2_ is studied in detail for pulsed laser deposition grown thin films on TiO_2_ substrates, under variation of temperature and applied bias currents. This system is of interest for novel electronics based on memristive concepts, in particular as the resistive transition in these films occurs close to room temperature. Multiple, stable resistance states can be set controllably in the temperature range of the hysteretic phase transition by tailored temperature sweeps or by Joule heating induced by current pulses.

## Introduction

Resistive switching and memristive-type devices are of interest for adaptable electronics and high density, low-power memory applications, including alternative computing paradigms such as in-memory computing and neuromorphic computing^1–9^. Of particular interest are systems in which, controllably, multiple resistive states can be achieved^6,10–13^. Resistive switching of the ‘valence-change mechanism’ type has typically been studied in metal-insulator-metal capacitor structures and reported in various binary and ternary metal oxides^1,3,14^. In these systems, ionic movement (mostly of oxygen vacancies) leads to electronic changes through formation of conducting filaments within the insulating matrix and/or by alteration of the transport properties of the interfaces between the oxides and the metal electrodes. Another important class of resistive switching devices is formed by the conducting bridge memristors, in which metallic filaments are emanating from one of the electrodes abutting an insulating dielectric^15^. In further configurations, involving e.g., insulating ferroelectric or magnetic layers, the electron transport can be controlled with the polarization state of these layers and the density of conducting domain walls^16–19^.

An attractive approach in the quest for memristive circuitry - circumventing the need for ionic transport - is the exploration of hysteretic metal-to-insulator (MIT) transitions in Mott insulators such as VO_2_ and NbO_2_^5,11,20^. In such Mott insulators, large resistance changes can be achieved e.g., through Joule heating near the MIT temperature or by carrier doping using electric field gating^5,21,22^. In particular vanadium-oxide (VO_2_) is an alluring resistive switching material, due to its very sharp, hysteretic MIT close to room temperature. Employing tailored temperature sweeps, multiple history-dependent resistance values can be defined^11,21,23–27^, as we will show below. Recently, Del Valle *et al*. have shown that the resistance values of vanadium-oxide films grown by RF magnetron sputtering on sapphire substrates can be tuned by electric field pulses, attributed to Joule heating effects when the pulses are applied at a temperature just below the MIT and by filamentary changes in the crystallographic configurations when the pulses are applied at considerably lower temperatures^25^. It is of interest to further explore this and to study the current- and temperature-induced changes in the resistance for the entire temperature range spanning the hysteretic metal-to-insulator transition and to explore in which ways multiple stable states can be induced. Both aspects are addressed here. In doing this, we also extend the range of deposition techniques and substrate materials.

## Methods

**Figure 1:**
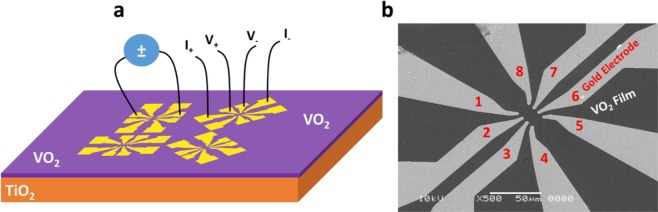
(**a**) Schematic of the device structure, representing a VO_2_ film grown on a TiO_2_ substrate, Au electrodes structured by photolithography, and the measurement geometry in two-probe and four probe configurations. (**b**) Corresponding scanning electron microscope image of these devices, showing the Au electrodes. The separation between contacts 1, 2, 3, 4 or 5, 6, 7, 8 is 3 µm, while these sets of contact are separated from each other by about 12 µm.

For our studies, epitaxial VO_2_ thin films showing a metal-to-insulator transition near room temperature were deposited on single crystal TiO_2_ (001) substrates using pulsed laser deposition, using a KrF excimer laser (λ = 248 nm, 20 ns pulse duration) and employing reflection high energy electron diffraction (RHEED) monitoring. The films were deposited at a temperature of 420 °C and in an oxygen background pressure of 1.2·10^−2^ mbar, by ablation from a V_2_O_5_ target with an energy density of ~1.3 J/cm^2^ and a pulse repetition rate of 2 Hz. After deposition, the samples were cooled at 10 °C/min at the same oxygen pressure. The high crystalline quality was verified using X-ray diffraction (XRD) (in both θ-2θ and reciprocal space mapping) and atomic force microscopy. A typical film thickness of ~10 nm was used, confirmed by X-ray reflectivity measurements. The electronic characteristics were measured in a four probe geometry, employing RF sputtered Ti-Au contacts defined by photolithography and lift-off, using the configuration shown in Fig. 1. Temperature-dependent measurements were performed with a temperature stability of approximately 50 mK.

**Figure 2:**
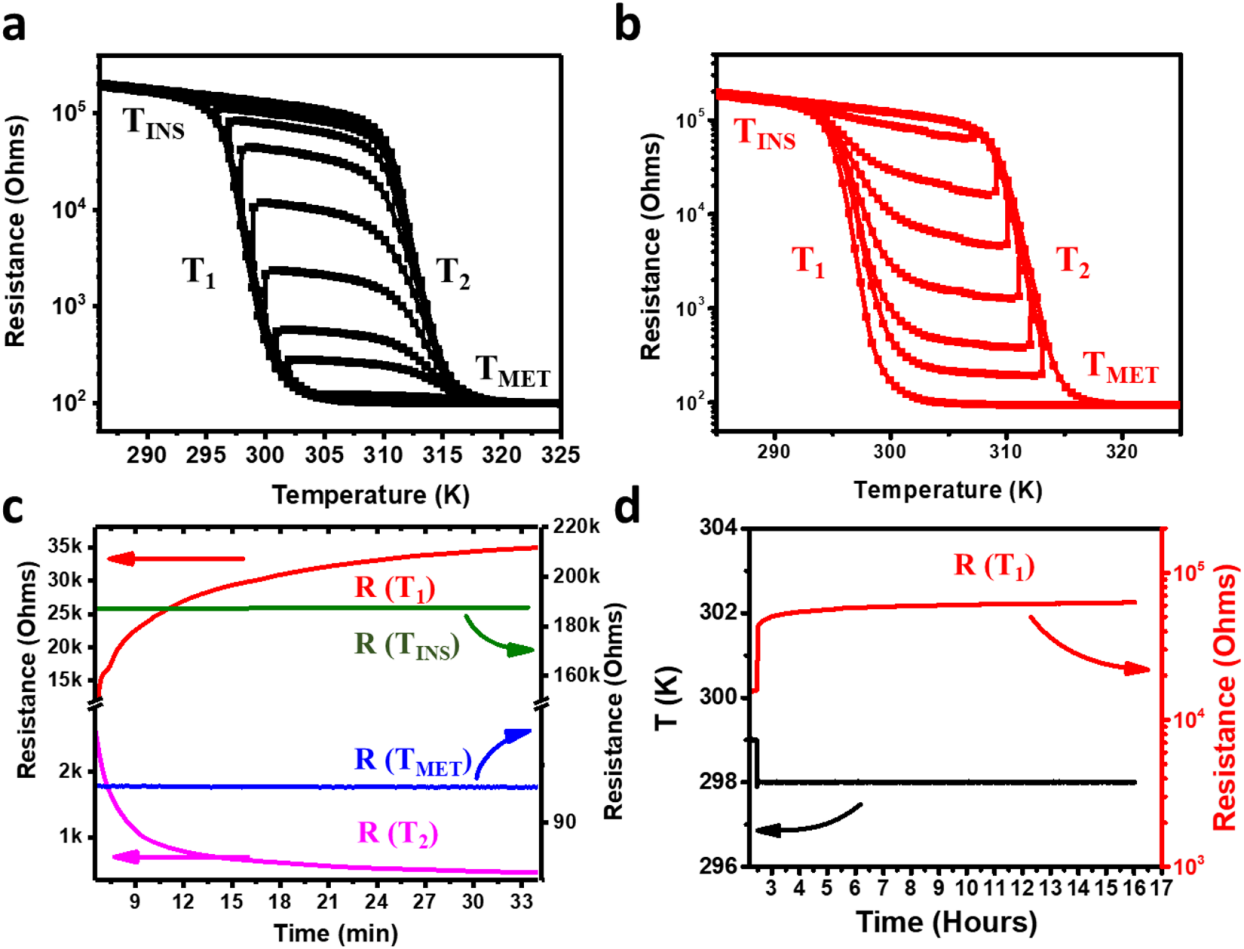
Multiple stable resistance states can be achieved when temperature sweeps are reversed. (**a**) Shows this effect when a cool down is reversed during the transition near T_1_, and (**b**) is for the case that warming up is reversed during the transition near T_2_. (**c**) Time-evolution of the different resistive states on a logarithmic scale showing the highly insulating state at T_INS_, the completely metallic state at T_MET_, and an intermediate resistance when keeping the temperature fixed at a given T_1_ during cooling or at a given T_2_ during heating. (**d**) The R(T_1_) on a linear scale vs longer time scale, showing the stability of the intermediate state after initial relaxation when switching the temperature from 299 K to 298 K.

The resistance *vs*. temperature *R(T)*, voltage *vs*. current *V(I)*, or current *vs*. voltage *I*(*V*) characteristics of the devices were studied while slowly scanning the temperature back and forth between 280 K and 360 K. A sharp metal-to-insulator transition is seen near 300 K when cooling the sample from high to low temperature, while the insulator-to-metal transition occurs near 315 K when heating the sample from low to high temperature (Fig. 2). In this manuscript, we will refer to these transition temperatures as *T*_1_ and *T*_2_, respectively. Further, we indicate with *T*_INS_ & *T*_MET_ temperature ranges at which the material is in a completely insulating or metallic state, respectively.

## Results and Discussion

Figure 2(a,b) shows the stable intermediate resistance states in the *R*(*T*) curves that were obtained when the temperature-sweep was reversed before completing the full transition, corroborating results obtained for sol-gel deposited films on sapphire^27^. After an initial relaxation, characterized by a long (*i.e*. several minutes) time scale (Fig. 2c), the intermediate resistance stabilized as shown in Fig. 2d. It is known that the overall metal-to-insulator transition coincides with a structural transition from a conducting rutile phase at high temperatures to an insulating monoclinic phase at low temperatures, concomitant with the opening of a band-gap in the band structure due to vanadium-vanadium dimerization^28,29^. In the hysteretic region there is a coexistence of metallic and insulating domains, as was seen in infrared microscopy^30,31^. If at a given intermediate resistance value within the MIT, the temperature is reversed and maintained between the limiting temperatures of the hysteresis loop for that resistance, the metallic puddle structure is stabilized^27^. This explains why in Fig. 2(a,b) the resistance remained roughly constant at the intermediate set-values when the temperature sweep was reversed at the corresponding point in the transition.

**Figure 3:**
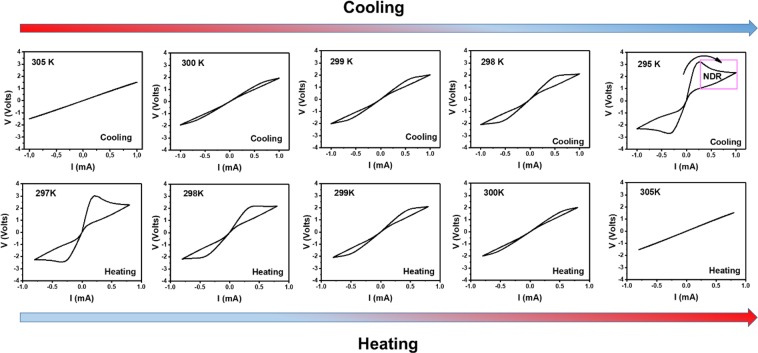
Voltage vs. current *V(I)* characteristics recorded at different temperatures while cooling from high temperature (top panels) or heating from low temperature (bottom panels).

**Figure 4:**
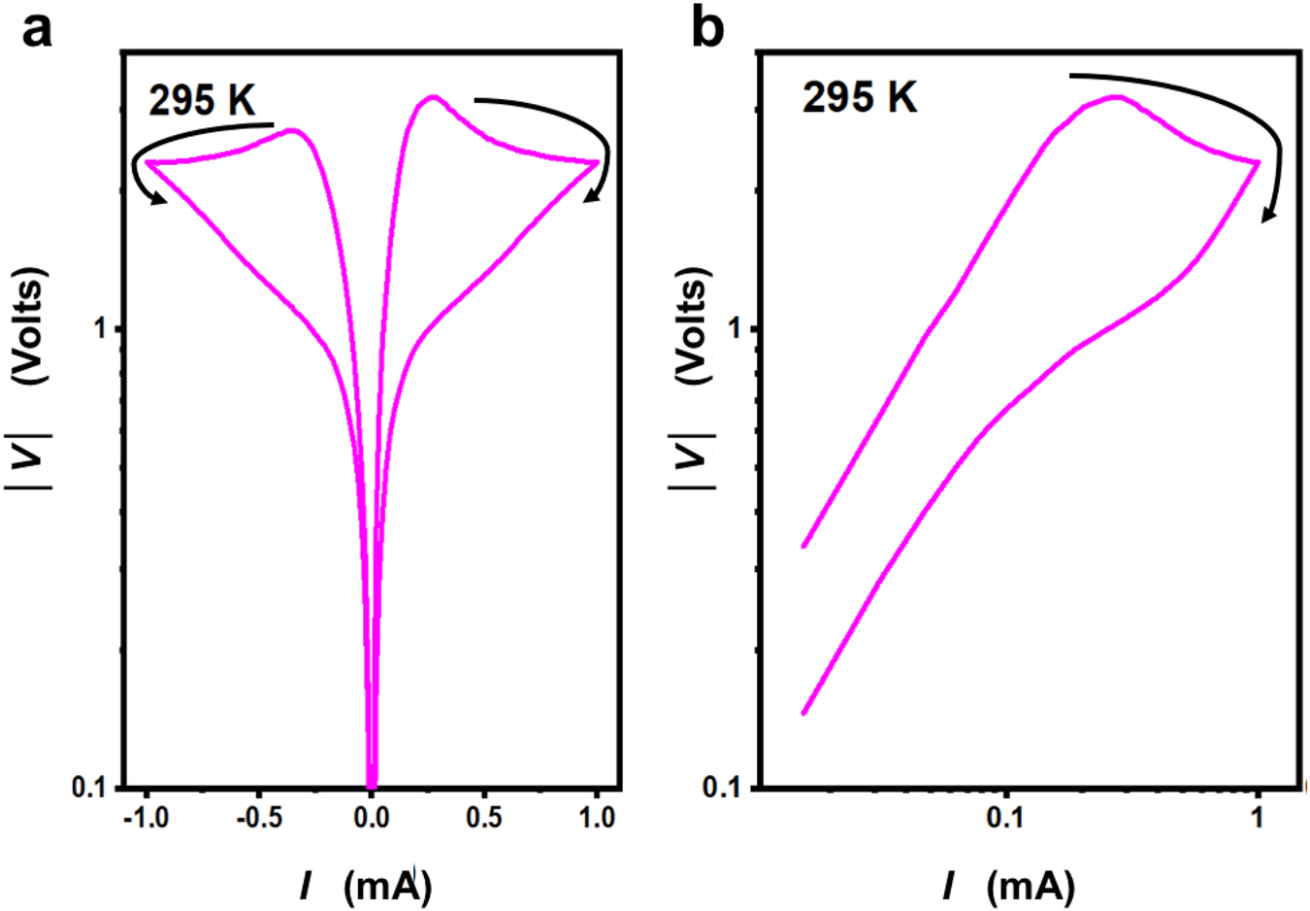
Voltage versus current *V(I)* characteristics at T = 295 K on (**a**) a log-linear scale and (**b**) a log-log scale.

The voltage *vs*. current *V*(*I*) characteristics show a qualitative development as a function of temperature as displayed in Fig. 3. At high temperatures, in the *T*_MET_ regime, Ohmic behavior is seen. When reducing temperature, in addition to the appearance of non-Ohmic behavior, hysteresis starts to occur systematically, which gets stronger as temperature is decreased. While on the linear scale of Fig. 3 it appears that the resistance around zero current/voltage has only a single value independent of the history of the current scan, a plot on a logarithmic scale (Fig. 4) reveals that the high and low resistive states actually stay distinct^23^. The *V*(*I*) characteristics entail a region with negative differential resistance (indicated in Fig. 3). It should be noted though that when reversing the current direction within this region, the *V(I)*-characteristics do not trace back along the same line, but close the hysteresis loop.

The resistive switching effects are symmetric for positive and negative current bias directions, as can be expected for a thermally driven process. When completing a sweep for one current bias polarity and repeating this without going first to the opposite, nearly the same trace was obtained, i.e. first a high resistance state was seen before the critical current was reached at which the resistance decreases. This implies that under these biasing conditions the system restores to a large extend to the higher resistance state near low bias. When measuring the *V*(*I*) characteristics starting at low temperatures and warming up, similar behavior was found as compared to measurements during cool down, as follows also from Fig. 3.

The *V(I)* characteristics of Figs. 3 and 4 were taken at temperatures close to the MIT. It is to be noted that the characteristics within this temperature range are different from the resistive switching observed at lower temperatures, further away from the MIT^32–36^. In the switching displayed in Figs. 3 and 4, electroforming and ion/defect migration are not expected to play a role. In line with this, the switching does not present a typical forming-set-reset loop and there is no need for opposite polarity to reset the switching. Also, switching is highly reversible with biasing and temperature, excluding all possibilities of permanent changes in the material or at interfaces. A more likely mechanism for the observed resistive switching is Joule heating, which can also lead to filamentary conductance paths^21,37^.

**Figure 5:**
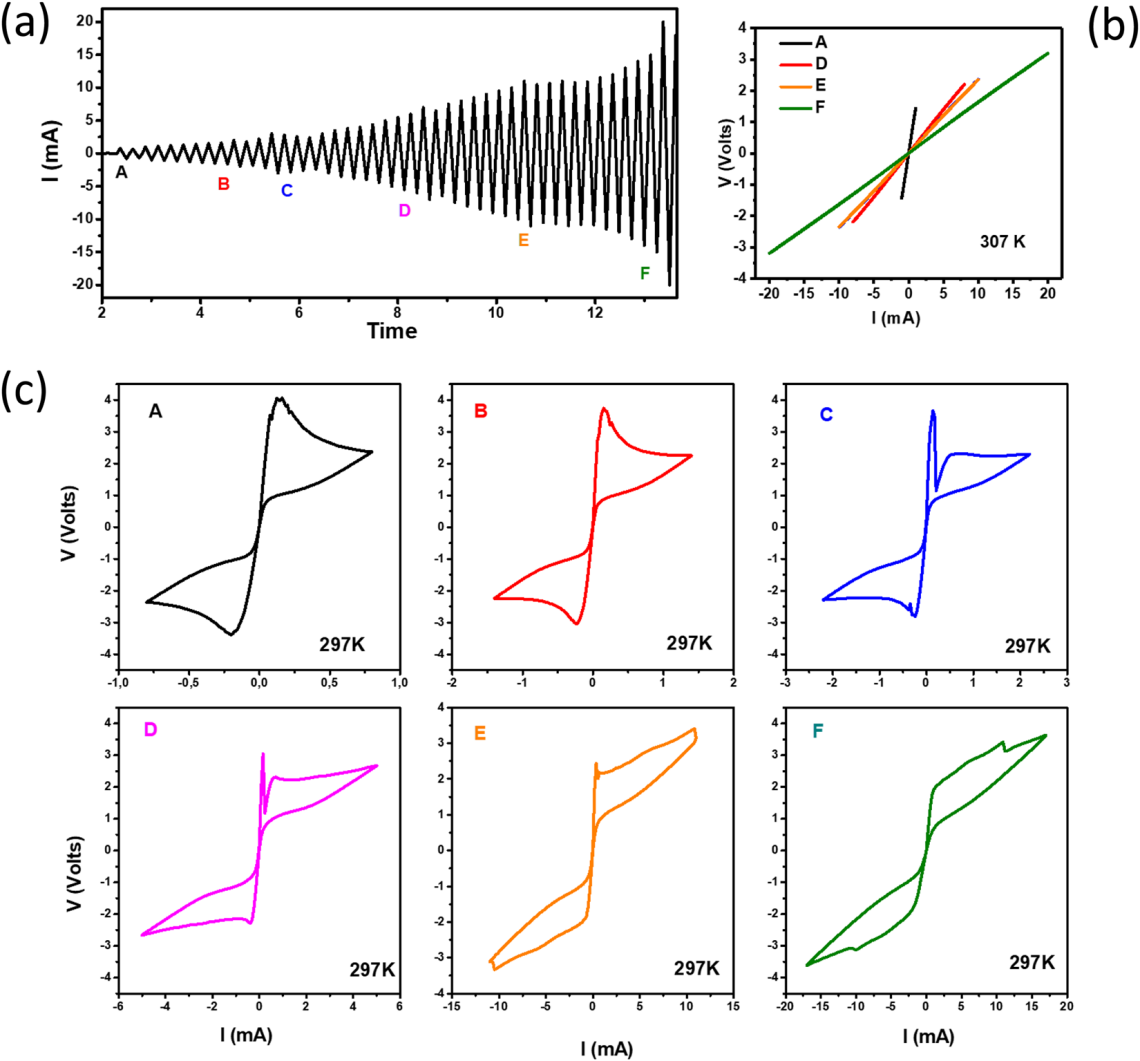
(**a**) Current bias applied to the sample; the labels A, B, C, D, E, F indicate the current range for each graph shown in figure (**b**) at 307 K and (**c**) at 297 K, respectively.

To study the bias-current dependence in more detail, the voltage was recorded for subsequent sweeps with varying return currents, as shown in Fig. 5. Figure 5(b) shows the behavior at 307 K, which is still within the MIT. The resistance changed each time when a higher current was applied. This memristive behavior is similar to that reported in^27^. Figure 5c shows the *V(I)* characteristics at a lower temperature (297 K). Clearly, as the current range is gradually increased, the heating effects lead to large and somewhat irregular hysteresis loops, suggesting more a avalanche-like formation and partial reconstruction of conducting filaments.

**Figure 6:**
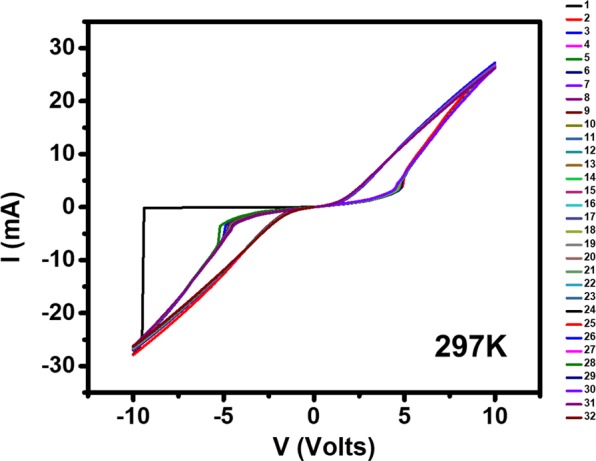
Thirty-two consecutive current vs. voltage *I(V)* characteristics (biasing the voltage 0 V → −10 V → + 10 V → 0 V) at 297 K. This temperature was set after cooling from 320 K.

To explore further, we have also obtained current *vs*. voltage *I*(*V)* characteristics at the comparatively low temperature of 297 K, shown in Fig. 6. Interestingly, a sharp jump in the first sweep was observed and all subsequent curves show similar hysteric behavior as was observed in Fig. 5. The first sweep, going down to −10 V, gives rise to a typical resistive forming step^21,35^. This sharp resistive switching could be exactly reproduced each time after fully cycling the temperature to above the MIT hysteresis region. Therefore, this switching is also due to heating effects, in accordance with^21^, and not due to defect migration.

## Conclusion

In conclusion, our studies underline that thermally induced phase transitions, either invoked by temperature sweeps or by current-induced Joule heating, provide useful pathways to create tunable resistors based on VO_2_. It should be noted though that the speed if this process is relatively slow, due to the inherent long time-scale of thermal processes and possible slow reconstruction processes within the sample after setting a certain resistance value. An advantage of the VO_2_ system over ionic migration-based devices is that by sweeping the temperature to above the metal-to-insulator transition, the written configuration can be completely erased. A prospect for future developments is the local writing of functional electronic circuits, including neuromorphic circuitry, by e.g. laser beams or heated AFM tips.

## Supplementary information

Supplementary information.
