# Supplementary material for: Resistive switching studies in VO2 thin films
Source: Sci Rep. 2020 Feb 24;10:3293. doi: 10.1038/s41598-020-60373-z (PMC7040009; doi:10.1038/s41598-020-60373-z)
Supplement: Supplementary file 1 — Supplementary information. [file 41598_2020_60373_MOESM1_ESM.docx]

**Supplementary Information**

**Resistive switching studies in VO_2_ thin films**

Abhimanyu Rana^1,2^, Chuan Li^1^, Gertjan Koster^1^, Hans Hilgenkamp*^1^

1. Faculty of Science and Technology, and MESA+ Institute of Nanotechnology, University of Twente, Enschede, The Netherlands
2. School of Engineering and Technology, BML Munjal University, Gurgaon, India

*: Corresponding author: H.Hilgenkamp@utwente.nl


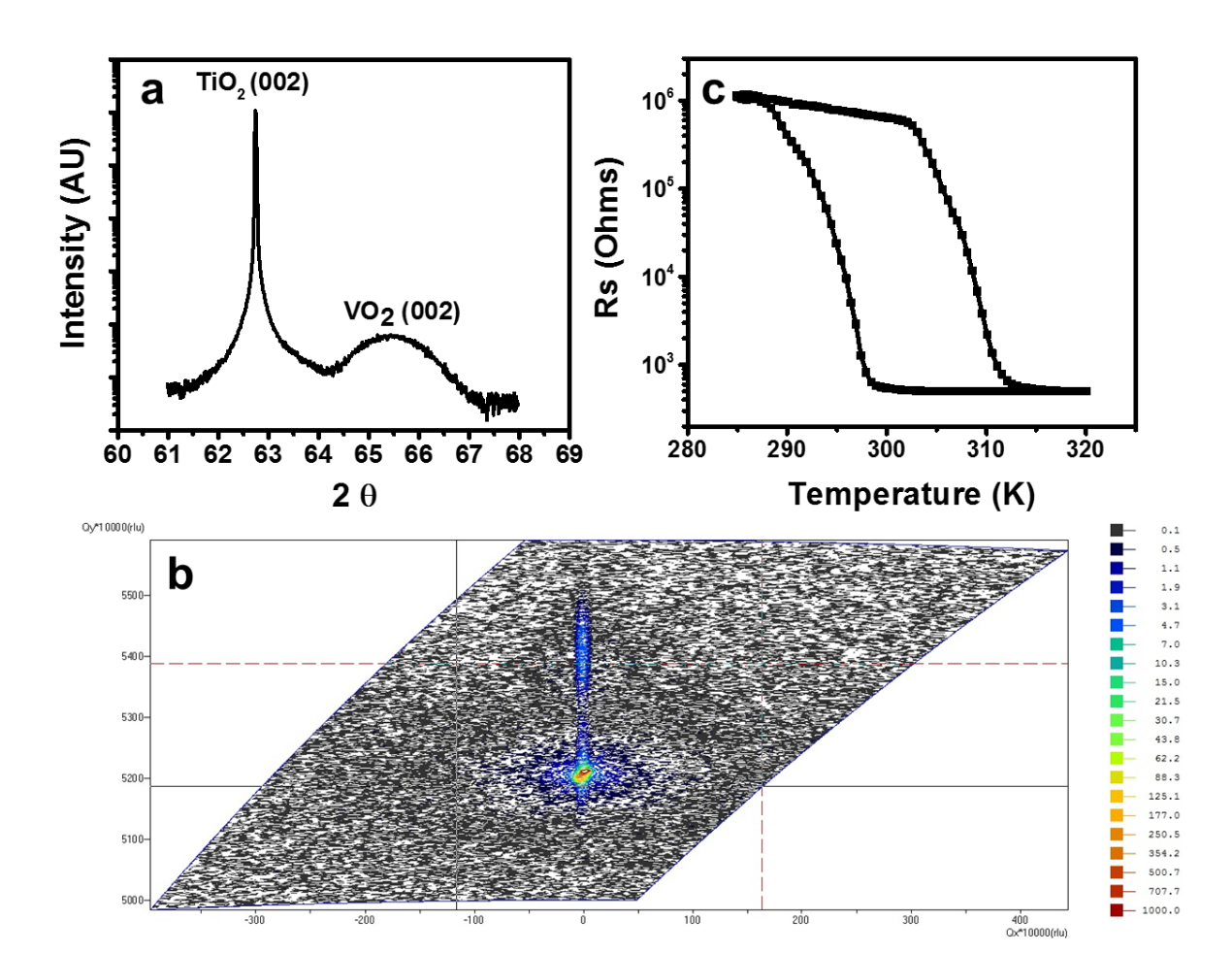


Figure S1: (a) shows the X-ray diffraction (XRD) 2θ scan of VO_2_ grown on a TiO_2_ (001) single crystal. (b) shows a XRD rocking curve (2θ-ω scan). The broad 2θ-peak (vertical axis) is consistent with the Scherrer formula giving a characteristic crystallite size of ~ 10 nm. However, the sharpness of the peak in the ω-scan (horizontal axis) indicates a high-quality smooth growth. (c) shows the sheet resistance versus temperature of full film measured in a van der Pauw geometry.


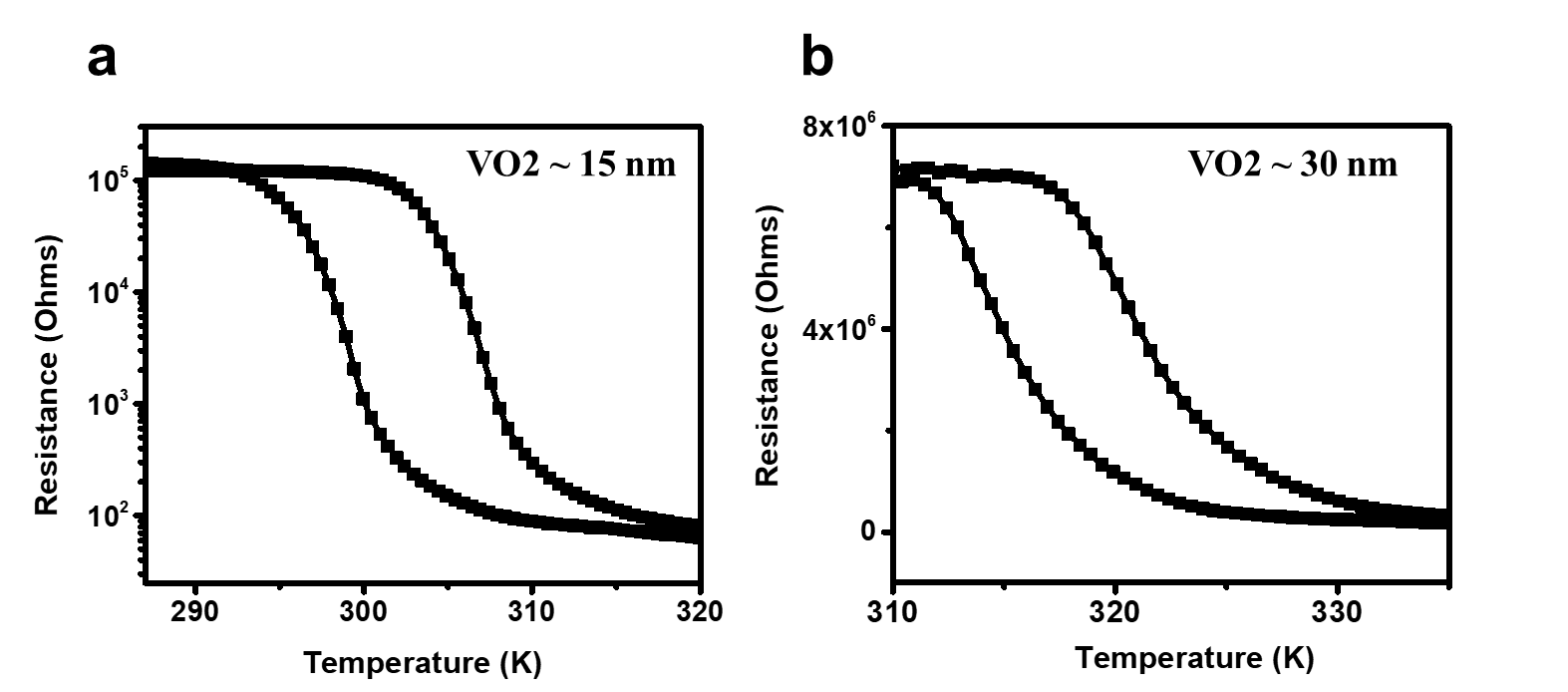


Figure S2: (a) & (b) show the resistance versus temperature of VO_2_ thin films of ~ 15 nm and ~ 30 nm thickness grown on TiO_2_ (001) substrates, indicating the thickness dependence of the insulator-to-metal transition temperature.


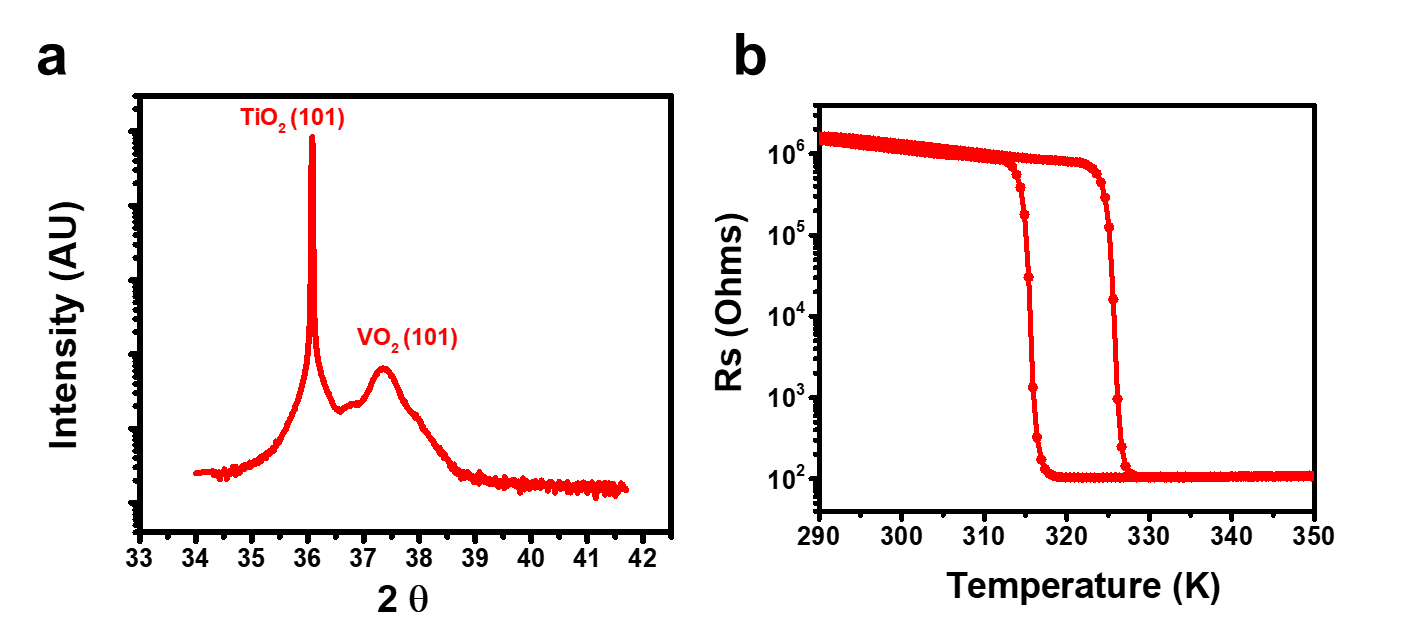


Figure S3: (a) shows the X-ray diffraction (XRD) results of VO_2_ grown on TiO_2_ (101) single crystal. These films are highly oriented to the (101) crystallographic direction. (b) shows the sheet resistance versus temperature of a (101)-oriented film measured in a van der Pauw geometry


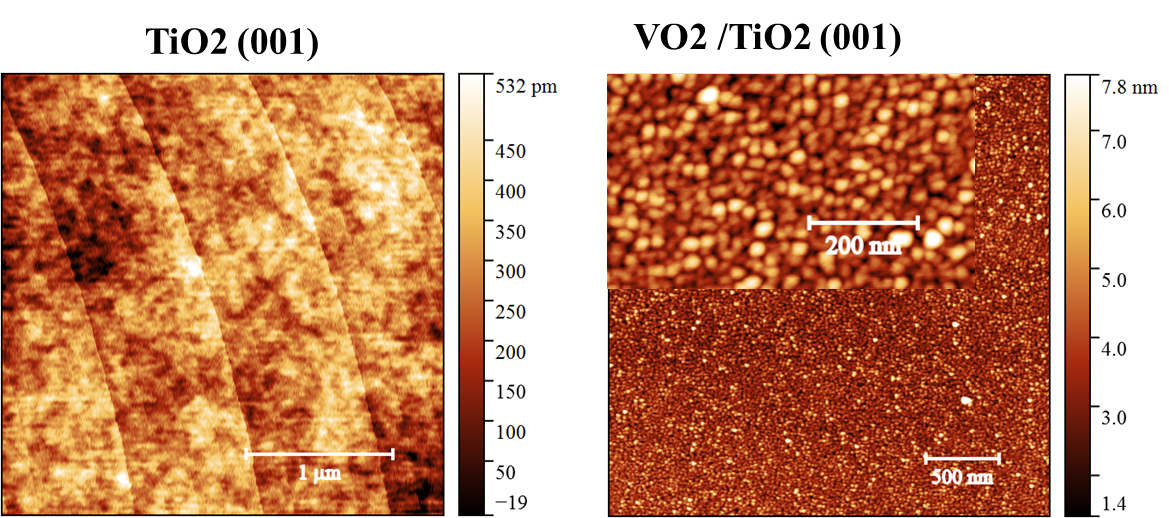


Figure S4: Atomic Force Microscopy (AFM) images of a TiO_2_ (001) single crystal substrate (a) before and (b) after the VO_2_ growth.


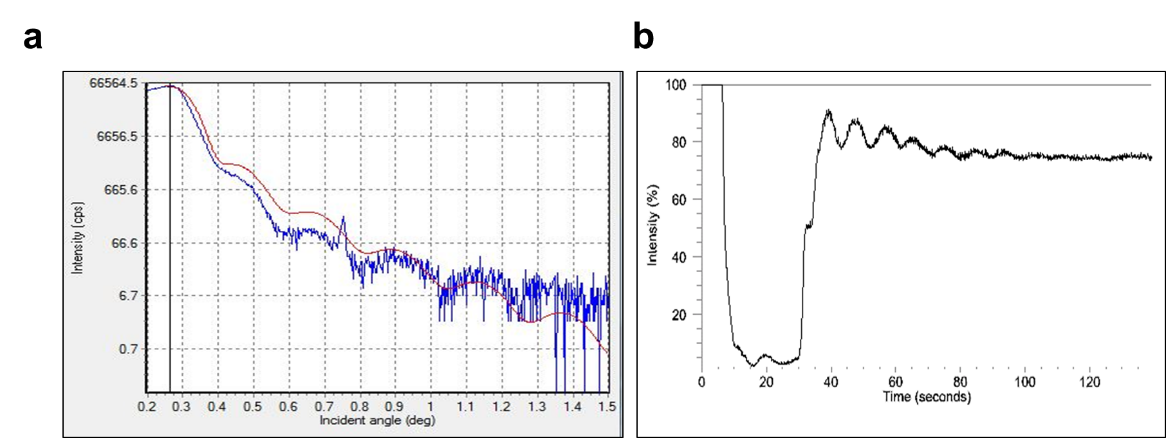


Figure S5: (a) shows an X-ray reflectivity measurement of VO_2_ grown on TiO_2_ (001) single crystal substrate. (b) displays the Reflection High Energy Electron Diffraction (RHEED) trace in pulsed laser deposition. Near 30 seconds the intensity was artificially increased to facilitate monitoring of the subsequent unit-cell oscillations during growth.
